# Adaptive Binarization of QR Code Images for Fast Automatic Sorting in Warehouse Systems

**DOI:** 10.3390/s19245466

**Published:** 2019-12-11

**Authors:** Rongjun Chen, Yongxing Yu, Xiansheng Xu, Leijun Wang, Huimin Zhao, Hong-Zhou Tan

**Affiliations:** 1 School of Computer Science, Guangdong Polytechnic Normal University, Guangzhou 510665, China; crj321@163.com (R.C.); permanyu@126.com (Y.Y.); xuxiansheng_fzy@163.com (X.X.); wangleijun@gpnu.edu.cn (L.W.); 2 School of Electronics and Information Technology, Sun Yat-Sen University, Guangzhou 510006, China

**Keywords:** QR code, automatic sorting system, uneven illumination, adaptive binarization

## Abstract

As the fundamental element of the Internet of Things, the QR code has become increasingly crucial for connecting online and offline services. Concerning e-commerce and logistics, we mainly focus on how to identify QR codes quickly and accurately. An adaptive binarization approach is proposed to solve the problem of uneven illumination in warehouse automatic sorting systems. Guided by cognitive modeling, we adaptively select the block window of the QR code for robust binarization under uneven illumination. The proposed method can eliminate the impact of uneven illumination of QR codes effectively whilst meeting the real-time needs in the automatic warehouse sorting. Experimental results have demonstrated the superiority of the proposed approach when benchmarked with several state-of-the-art methods.

## 1. Introduction

**Figure 1:**
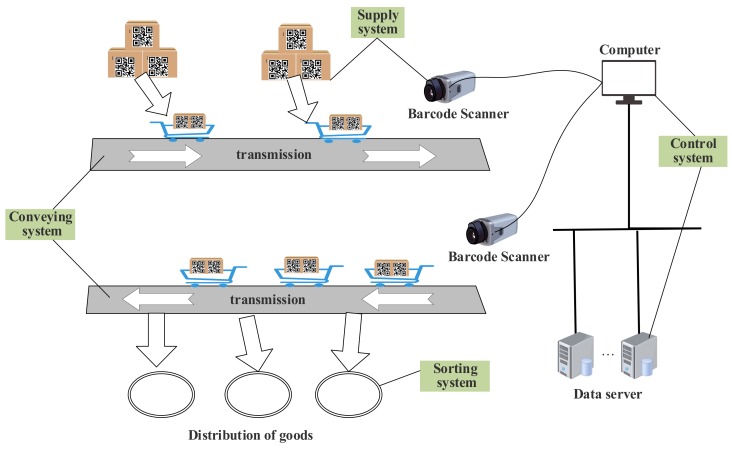
Diagram of warehouse automatic sorting system.

It is estimated that over 50 billion devices will be connected to the Internet by 2020. Accompanying the increasing popularity and wide applications of the Internet of Things (IoT), it will affect all areas of industry, including smart homes, advanced manufacturing, intelligent transportation and healthcare. The IoT can make a connection between the physical world and the virtual world through sensors, automatic identification, embedded systems and other technologies, which has become a new trend for the future Internet [1,2,3,4]. Moreover, the automatic identification technology represented by two-dimensional code is one of the most critical technologies in the IoT, which provides an entrance for the connection between the object and the network. Furthermore, the QR code is one of the most widely used two-dimensional bar codes, which has the advantages of large information capacity, strong error robustness, and low cost [5]. Since there are three position detection patterns with the same width ratio of the fixed module, it can be easily recognized at any viewing angle [6]. Therefore, the QR code is widely used to manage warehouse logistics. Herein, the main facility responsible for management is the automatic sorting system [7]. Under the coordination of the control system, the supply system sends the barcode-affixed goods to the sorting system via the conveying equipment. The sorting equipment obtains the predetermined result according to the cargo information to classify the goods accordingly. The workflow diagram of the automatic sorting system is shown in Figure 1.

Since the automatic sorting system sorts the goods according to the associated QR code, rapid and accurate classification of the QR code is the key to the supply system. How to identify the QR code quickly and accurately depends on the efficacy of the sorting system, as well as the capacity and scale of the warehouse. However, the complex and variable lighting environment often leads to uneven illumination of the QR code. Such nonuniform illumination affects the binarization and makes it difficult or inaccurate in identifying the QR code quickly and accurately. Thus, it adds much unnecessary time cost to warehouse management systems.

To address this challenging issue, many attempts have been proposed in recent years. Di et al. developed an improved local Wellner’s algorithm to identify the QR code label on the package in the auto-sorting system [8]. The principle of the algorithm is simple, while it will spend more time in processing, and the effect of processing the unevenly illuminated QR code images in complex lighting environments is far from the best. Yao et al. presented an algorithm that combines the improved Niblack’s algorithm with the Otsu’s algorithm [9]. The algorithm is faster but does not have good adaptive ability as it fails to adapt to the different uneven illumination of QR code images. Duan et al. put forward a method suitable for nonuniform illumination bar codes [10], but the algorithm is more complex and does not handle well the boundaries of uneven lighting. Wu et al. came up with a method that separates the QR code into several small blocks by block truncation coding (BTC) [11], but the steps of the algorithm are lengthy and the results are sensitive to the size of the block window. Yang et al. introduced an improved local threshold method based on the Bernsen’s method [12], but the parameters need to be determined manually.

Therefore, we propose a fast-adaptive thresholding method based on symbol features of the QR code for further improvement toward practical expectations from the industry. It solves the issues of long-time cost, poor adaptive ability, and low robustness. The method takes the size of the dark module in the middle of the position detection patterns of the QR code as the selected image block and divides the QR code image into two parts by the window and processes adaptively as guided by the cognitive mechanism. The experimental results illustrate that the proposed approach has a strong adaptive ability, better processing speed, and higher detection accuracy than several existing methods.

The rest of the paper is organized as follows: Section 2 mainly introduces the related work in available threshold techniques. Section 3 presents the details of the proposed approach. The experimental results are given and compared in Section 4, including benchmarking with other approaches and also extended performance analysis under different testing conditions. Finally, Section 5 concludes the paper.

## 2. Related Work

We explain the basic concept of image binarization and analyze the available threshold techniques.

It is generally accepted that image binarization is one of the most commonly used image processing methods [13]. Many binarization techniques in processing tasks are aimed at simplifying and unifying the image data at hand. As a particular image, the QR code comprises black and white modules, with the white module as the background and the black module containing the information. The basic idea of image segmentation is to select the proper threshold to set the gray value of each pixel of the image to 0 or 255. Thus, the whole image shows a distinct black and white effect. The process assumes the grayscale value of each pixel is $f\left(x,y\right)$, and the threshold is T, whose values from 0 to 255, then:

(1) $$g\left(x,y\right)=\left\{\begin{array}{c} \;\;\;0\;\;\;\;f\left(x,y\right)\leq T\\ 255\;\;f\left(x,y\right)>T \end{array}\right.$$

where $g\left(x,y\right)$ denotes the gray value of each pixel in the binarized image. The pixel shows that white, when $g\left(x,y\right)$ is 255, while it is black when $g\left(x,y\right)$ is 0. Depending on the definition of thresholds, the methods of binarization can be divided into two categories, including the global threshold method and the local threshold method [14]. The principle of the global threshold method is easy to understand, such as the Otsu’s algorithm [15], a typical method of global thresholding, but performing binarization of QR code in a complex lighting background is poor. Compared with the global threshold algorithm, the effect of the local threshold method has better performance and the target feature of the image can be extracted from the complex lighting environment. Among the local threshold methods, the Niblack’s algorithm [16] is a more commonly used method. However, there will be discontinuity and a more obvious block boundary effect in the sub-block images generated by the local threshold methods. What’s worse, the local threshold methods exhibited more considerable computation and higher complexity.

The traditional local threshold algorithms are not effective in dividing the nonuniform illumination image, and the calculation time is too long. Consequently, many scholars have put forward lots of improved algorithms to solve the problems. Wu proposed a method using a block truncation coding (BTC) to divide an image into a plurality of blocks but selecting the algorithm block window size is arbitrary [11]. Even if the same size of the block window is selected to divide various uneven light images, the processing effect also will be substantially different. Zineb et al. developed an improved local threshold method, whose parameters of block window are defined for the whole image [17]. Thus, it cannot be distinguished well for the segmentation of small targets and large targets in the same image, which is hard to find the fitting block window. Literature [18] proposed an improved algorithm based on the Sauvola’s algorithm, however, the algorithm only improves the way of deciding the threshold, which does not change the means of determining the size of the image block window. The adaptive ability of the algorithm, therefore, is not good enough and there is still much room for improvement. Saddami et al. proposed a new binarization method using a novel local adaptive threshold to extract information from nonuniform illumination images [19]. The new local threshold is an adaptive mean value. Nevertheless, the most appropriate local window size is a fixed value, which is difficult to obtain from experiments. Lu et al. proposed an image binarization algorithm using a quadtree to divide areas adaptively [20]. It is particularly useful for severely uneven illumination images; however, a high computational cost is incurred due to the complexity between image regions. Literature [21] proposed a novel adaptive region-wise histogram correction and thresholding technique, which works well for images with poor lighting quality, but the number of sub-images in the local threshold binarization process is unspecified clearly.

Taken from the analysis above, we conclude that the current improved algorithms find it difficult to determine the size and quantity of block windows or block areas suitably. There will be discontinuities between blocks and blocks or regions and regions after binarizing the image. When the size of the uneven illumination image or the degree of uneven light is changed, the result will have a vast difference, resulting in weak adaptive ability. Hence, we have created the approach combining the features of the QR code image to find the most suitable block window.

## 3. Proposed Method

According to the characteristics of the QR code symbol, the proposed method selects the size of the block window adaptively and then uses the window to divide the uneven illumination of the QR code image into several blocks. The following step is to perform threshold processing on each block image and then combine them in sequence. Finally, the complete QR code is reconstructed to achieve the binarization of the nonuniformly illuminated QR code image.

### 3.1. Preprocessing of QR Codes

Since the block window is closely related to the size of the position detection pattern, the proposed algorithm enhances the contrast of the QR code image to ensure the image finds the position detection pattern after preliminary binarization. The contrast of uneven illumination of the QR code is enhanced by the bottom hat transform, followed by the transformed image being preprocessed by the variable threshold algorithm based on local statistics.

The bottom hat transform is used to extract bright features from the darker background [22], then the position detection pattern can be extracted from the uneven illumination of the QR code. The process of the bottom hat transform of a grayscale image I is:

(2) $$B_{hat}\left(I\right)=\left(I⊗b\right)-I$$

where ⊗ represents the morphological closing using the structural element b, and b is defined as follows:

(3) $$b=\frac{\max\left(h,w\right)}{7}$$

where h and w are the height and width of the QR code image, according to the ratio of 1:1:3:1:1 in position detection patterns of the QR code, the denominator is set to 7.

The basic method of the local threshold is to calculate the standard deviation $\sigma_{xy}$ and mean value $m_{xy}$ of the pixels around the pixel point and then to determine the threshold. The general form is:

(4) $$T_{xy}=c_{1}\sigma_{xy}+c_{2}m_{xy}$$

where $c_{1}$ and $c_{2}$ are non-negative constants. Based on our experiments, we found that $c_{1}$ can be an adaptive value in the range of 1–30, and the most appropriate value is 3, while $c_{2}$ is a fixed value which equals to 1.

**Figure 2:**
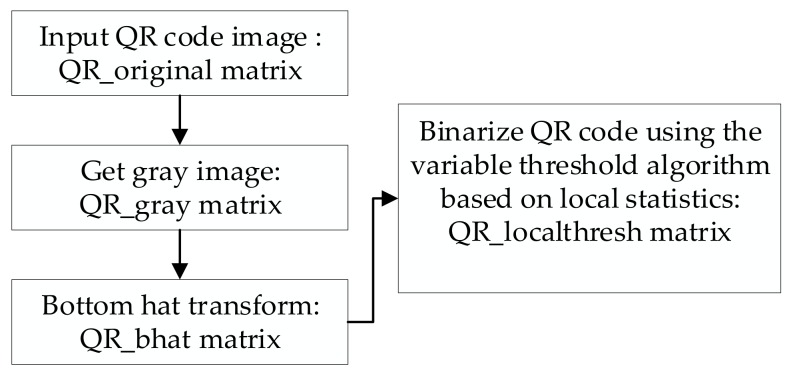
Diagram of preprocessing.

The variable threshold algorithm based on local statistics is based on local threshold processing, which combines local characteristics by logic rather than arithmetic [23]. Its definition is:

(5) $$g\left(x,y\right)=\left\{\begin{array}{c} 1\;f\left(x,y\right)>c_{1}\sigma_{xy}\;AND\;f\left(x,y\right)>c_{2}m_{xy}\\ 0\;other \end{array}\right.$$

where $g\left(x,y\right)$ represents the grayscale value of pixels in a binarized image. $f\left(x,y\right)$ is the input image, and $m_{xy}$ is the mean of the image. A schematic diagram of the preprocessing is shown in Figure 2.

### 3.2. Adaptive Selection of Block Window

After the preprocessing of the unevenly illuminated QR code image, the position detection patterns can be observed. It has a unique proportion among modules, in the area, which is 1:1:3:1:1 and exists in horizontal, vertical and slanting directions, while the data and error correction code words in the QR code do not exhibit the specific ratio due to the masking operation.

#### 3.2.1. Selection Process of Adaptive Block Window

**Figure 3:**
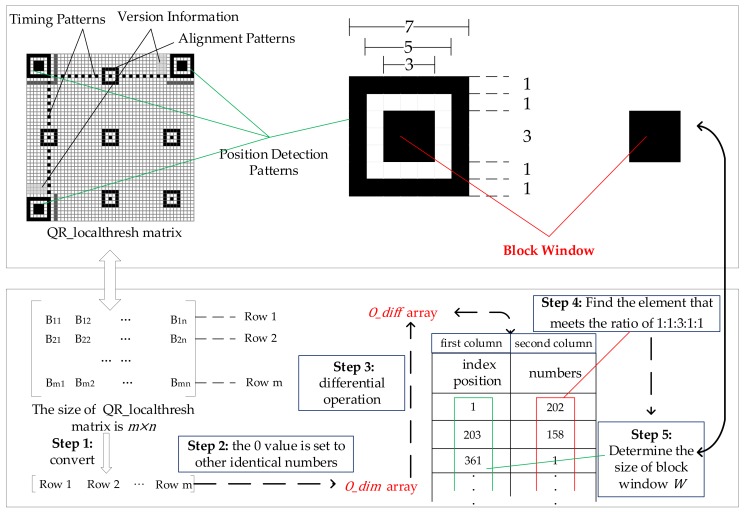
Process of the adaptive selection of the block window.

We first detect the position detection patterns of QR code by the proportional characteristics, in the proposed method. Then, the algorithm determines the size of the block window based on the position detection patterns. The elaborated illustrations of the adaptive selecting block window are shown in Figure 3.

(1) During the above process, it is necessary to convert the matrix of the preprocessed QR code image to a one-dimensional array, O_dim, in the order of rows, and the value equal to zero in O_dim is set to other identical numbers to facilitate the subsequent differential operation.
(2) Perform a differential operation on the O_dim and record the obtained array with two columns as O_diff, where the second column data of O_diff represents the number of consecutive identical elements in the O_dim. The first column of data matches the starting position in the O_dim, which is defined as the index position.
(3) Find the element that meets the ratio of 1:1:3:1:1 based on the last column data of O_diff, then calculate the size of the dark module in the middle of the position detection pattern by the index position corresponding to the element in the first column data. Namely, the index position difference corresponding to the proportional value 3 is equal to the side length of the dark module. Accordingly, the algorithm selects the size of the block window W to be w×w adaptively.

#### 3.2.2. Analysis of the Feasibility Principle of Adaptive Block Window

The paper corroborates the rationality and robustness of the proposed algorithm to select the block windows from two aspects. We tested the effect on the selection of different windows to binarize different sizes of QR code with the same type of uneven illumination conditions, for one thing. We also tested the effect selecting different uneven illumination QR codes of fixed size for binarization, for another. Moreover, the binarized images are evaluated by full reference objective quality metrics, including peak signal-to-noise ratio (PSNR) and structural similarity (SSIM).

PSNR is most simply defined via mean square error (MSE). Given an m×n monochrome image I and its approximate image *K*, the MSE and PSNR can be defined as:

(6) $$MSE=\frac{1}{mn}\overset{m\;-\;1}{\underset{i\;=\;0}{\sum }}\overset{n\;-\;1}{\underset{j\;=\;0}{\sum }}{\left[I\left(i,j\right)-K\left(i,j\right)\right]}^{2}$$

(7) $$PSNR=10\;\cdot \;log_{10}\left(\frac{MAX_{I}^{2}}{MSE}\right)$$

Concerning Equations (2) and (3), $I\left(x,y\right)$ and $K\left(x,y\right)$ are the corresponding gray value or color value in the original image and the reconstructed image, respectively [24]. Considering the broad dynamic range, PSNR is usually expressed in the logarithmic decibel scale. Here, $MAX_{I}$ is the maximum pixel value of the image. Generally,$\;MAX_{I}\;$ is $2^{B}-\;1$. B is the color depth, which means several binary digits represent one pixel, and B is often taken as 8.

Unlike PSNR, SSIM is a method to compare the correlation between the distortion image and the reference image directly. Given two images x and y, the structural similarity is defined as:

(8) $$SSIM\left(x,y\right)={\left[l\left(x,y\right)\right]}^{\alpha }{\left[c\left(x,y\right)\right]}^{\beta }{\left[s\left(x,y\right)\right]}^{\gamma }$$

where $l\left(x,y\right)$,$\;c\left(x,y\right)$ and $s\left(x,y\right)$ are the comparison functions of luminance, contrast and structure, respectively. The definition is as follows [25]:

(9) $$l\left(x,y\right)=\frac{2\mu_{x}\mu_{y}+C_{1}}{\mu_{x}^{2}+\mu_{y}^{2}+C_{1}},c\left(x,y\right)=\frac{2\sigma_{x}\sigma_{y}+C_{2}}{\sigma_{x}^{2}+\sigma_{y}^{2}+C_{2}},s\left(x,y\right)=\frac{\sigma_{xy}+C_{3}}{\sigma_{x}\sigma_{y}+C_{3}}$$

The weights α, β and γ are greater than 0, which are parameters for adjusting the relative importance of the three comparison functions.$\;\mu_{x}$ and $\mu_{y}$ are the average of the images x and y. $\sigma_{x}$ and $\sigma_{y}$ are their standard deviations. $\sigma_{xy}$ denotes the covariance of two images. $C_{1}$,$\;C_{2}$ and $C_{3}$ are the constants to maintain the stability of the comparison functions.

**Figure 4:**
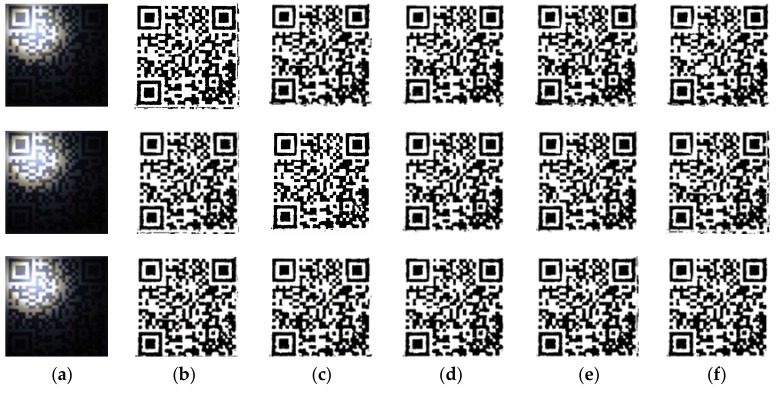
The experimental results in ascending order of image size. (**a**) Original image; (**b**) Block size w – 10; (**c**) Block size w  – 5; (**d**) Block size w; (**e**) Block size w  + 5; (**f**) Block size w  + 10.

**Figure 5:**
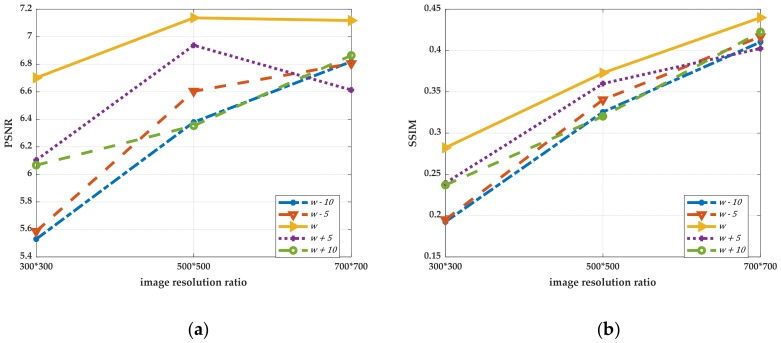
The values of PSNR and SSIM of different sizes of QR codes under different windows processing. (**a**) The line graph corresponding to PSNR; (**b**) The line graph corresponding to SSIM.

**Figure 6:**
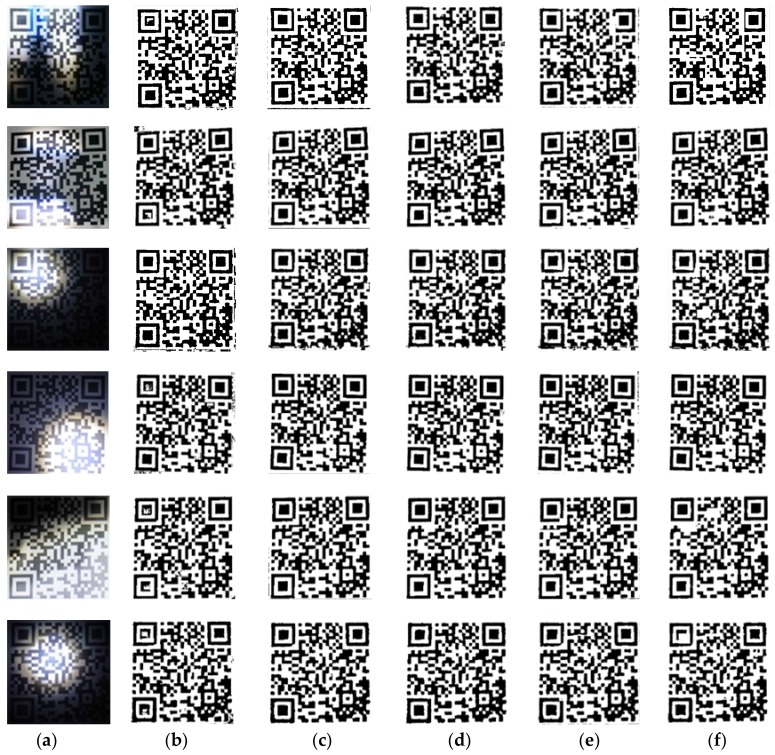
The experimental results of fixed image size. (**a**) Original image; (**b**) Block size w – 10; (**c**) Block size w  – 5; (**d**) Block size w; (**e**) Block size w  + 5; (**f**) Block size w + 10.

**Figure 7:**
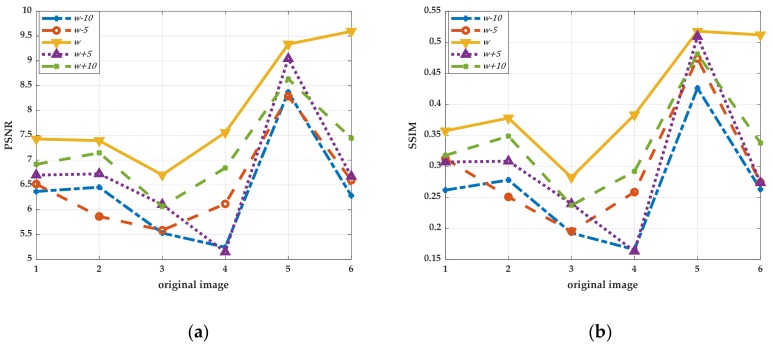
The values of PSNR and SSIM of QR codes in the same size under different window processing. (**a**) The line graph corresponding to PSNR; (**b**) The line graph corresponding to SSIM.

The algorithm obtains the window size as w×w adaptively. The comparison experiments are performed by increasing and decreasing w to change the size of the block window. We selected one group of uneven illumination QR code images for experiments. This group of experiments only changed the resolution of images, in which the images with resolutions of 300 pixels × 300 pixels, 500 pixels × 500 pixels and 700 pixels × 700 pixels were selected as the experimental objects. The results in ascending order of image size are shown in Figure 4, and the line graphs corresponding to PSNR and SSIM values are shown in Figure 5. Then, we selected six kinds of unevenly illuminated QR code images for experiments, the resolution of which was 300 pixels × 300 pixels. The experimental results are shown in Figure 6 and the values corresponding to the PSNR and SSIM indicators are shown in Figure 7.

The experimental results in Figure 4 demonstrate that even when the size of the same kind of uneven illumination of the QR code is changed, the effect of binarization is still the best with the same window size of w×w. Moreover, the corresponding PSNR and SSIM values are also the largest. While changing the size of w to split and binarize the QR code, the image will lose a part of the information and produce more noise, and the corresponding PSNR and SSIM will decrease definitely. The analyses of the experimental results in Figure 6 and Figure 7 reveal that different types of uneven illumination QR codes with the same size have the best performance of binarization when the window size is w×w, which means that the QR code contains more information. After changing the size of the window, the QR code reconstructed will generate more noise and the values of the PSNR and SSIM indicators will be correspondingly smaller.

The conclusion can be drawn from the experiments that the proposed algorithm is reasonable in the size selection of the block window, since the block window is closely related to the QR code, which results in the strong adaptive ability of the approach. Even if the size of the unevenly illuminated QR code image is changed, or different types of unevenly illuminated QR code images are selected, the algorithm can find the most suitable block window quickly. More importantly, the method offers a standard for the size selection of the block window, avoiding the arbitrariness and irrationality of window selection, and simultaneously improves the processing speed. Furthermore, in the case where the experimental effect allows, the size selection of the block window can be a range. As shown in the above experiments, when the window size length is w∈w±10, the proposed algorithm also can have a better effect of binarization. It also confirms that the proposed method has better robustness in the selection of the block window.

### 3.3. Binarization of QR Codes

**Figure 8:**
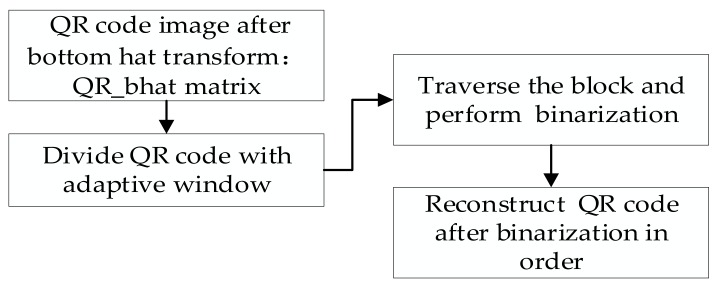
Process of QR code binarization.

The detailed process of binarization is to use the window selected adaptively to divide the QR code image. Then, each piece of image is binarized by the Otsu method and combined sequentially to reconstruct the QR code. The method can eliminate the impact of the uneven illumination of the QR code effectively and achieve a better effect of binarization rapidly. The flow diagram of binarization is shown in Figure 8.

**Figure 9:**
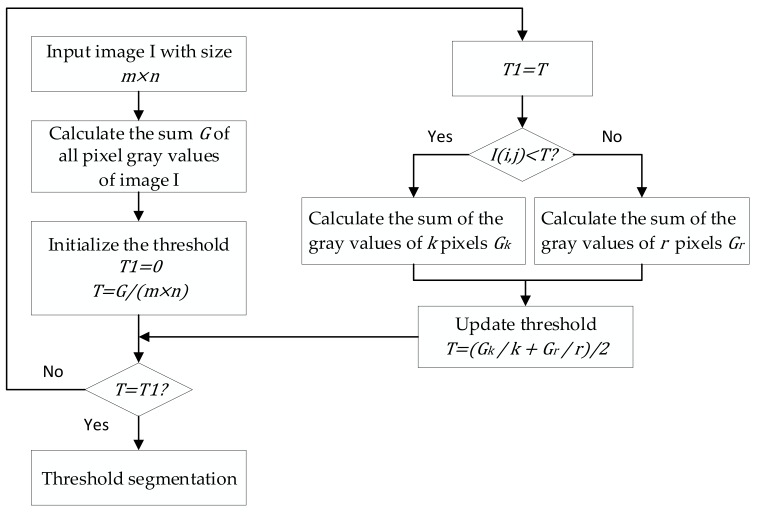
Process of cyclic threshold algorithm.

(1) The principle of using window w to split the QR code image after the bottom hat transform is as follows. The QR code image after the bottom hat transform is divided into blocks from left to right. When the total length of all the blocks exceeds the image width, the QR code is at most divided into the image height. Likewise, for the QR code image after the bottom hat transform is divided into blocks from top to bottom. When the total length of all the blocks exceeds the image height, the QR code is at most divided into the image width.
(2) All the blocks are traversed, and each block is binarized by the Otsu’s method. However, it was found in the experiment that the image information at the first block location sometimes disappears. Through analysis, we found that the first block image contains an indefinite foreground and background after enhancing the contrast of the uneven illumination of the QR code. Therefore, the Otsu’s algorithm cannot obtain a better binarization effect after processing the image. The defect can be eliminated when the cyclic threshold algorithm processes the first block. The schematic diagram is shown in Figure 9.
(3) All binarized QR code blocks are combined sequentially to reconstruct a whole QR code image.

## 4. Experiments

The experimental settings have a significant influence on the recognition of the QR code. The higher the accuracy of the equipment used to collect the image, the better the effect of the algorithm. The experimental conditions can be divided into hardware equipment, software environment, and related experimental materials as shown in Table 1, Table 2 and Table 3, respectively. Moreover, each set of images collected with a mobile phone is uniformly processed using the popular image editing tool Adobe Photoshop to make them equal in sizes. Then, calculate the peak signal-to-noise ratio (PSNR) and structural similarity (SSIM) between the QR code image processed by different algorithms and the original image of the nonuniformly illuminated QR code. Concerning the experiment of the QR code recognition rate, first, the binarization images processed by different algorithms are printed, which are recognized by the barcode recognition software ZXing AndroidSDK and WeChat. Then, we count the recognition rates of different algorithms to complete the experiment.

We selected five types of unevenly illuminated QR code images for testing, which showed the advantages of the proposed method in dealing with the uneven illumination of QR codes. The experimental effect of other algorithms is compared with the proposed algorithm, and the PSNR and SSIM indexes are used to evaluate the image quality after binarization.

**Figure 10:**
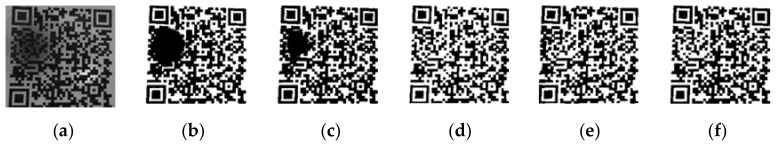
The original image and experimental results from different algorithms: (**a**) Original image; (**b**) Otsu’s algorithm; (**c**) Niblack’s algorithm; (**d**) Yao’s algorithm; (**e**) Di’s algorithm; (**f**) Proposed method.

**Figure 11:**
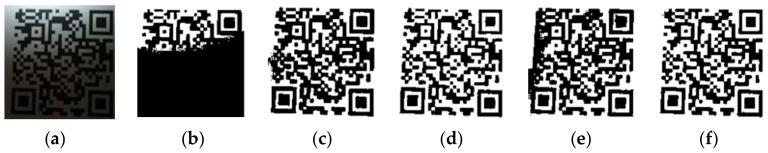
The original image and experimental results from different algorithms: (**a**) Original image; (**b**) Otsu’s algorithm; (**c**) Niblack’s algorithm; (**d**) Yao’s algorithm; (**e**) Di’s algorithm; (**f**) Proposed method.

**Figure 12:**
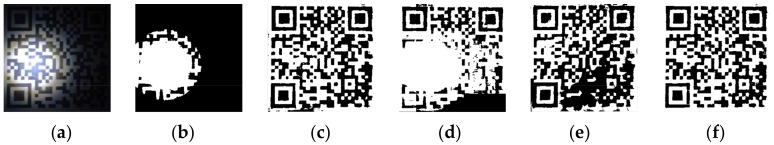
The original image and experimental results from different algorithms: (**a**) Original image; (**b**) Otsu’s algorithm; (**c**) Niblack’s algorithm; (**d**) Yao’s algorithm; (**e**) Di’s algorithm; (**f**) Proposed method.

**Figure 13:**
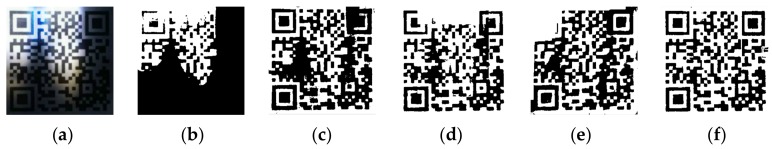
The original image and experimental results from different algorithms: (**a**) Original image; (**b**) Otsu’s algorithm; (**c**) Niblack’s algorithm; (**d**) Yao’s algorithm; (**e**) Di’s algorithm; (**f**) Proposed method.

**Figure 14:**
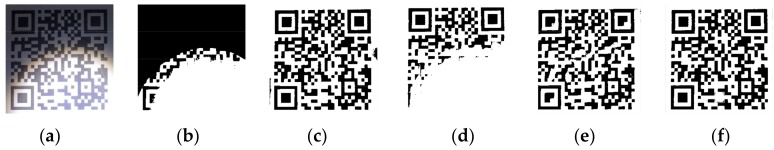
The original image and experimental results from different algorithms: (**a**) Original image; (**b**) Otsu’s algorithm; (**c**) Niblack’s algorithm; (**d**) Yao’s algorithm; (**e**) Di’s algorithm; (**f**) Proposed method.

(1) The effect of different algorithms in dealing with the uneven illumination of the QR code in Di’s paper [8] is shown in Figure 10. The reason why the phenomenon of uneven illumination occurs is lack of light in the acquisition resulting in a low local gray value of the image.
(2) The effect of different algorithms in dealing with the uneven illumination of the QR code in Yao’s paper [9] is shown in Figure 11. The result of this uneven lighting phenomenon is due to the collection of light which is dark, or the limited acquisition by the device so that the overall gray value of the image is low.
(3) The effect of different algorithms in dealing with uneven QR codes with strong light spots is shown in Figure 12. The local light intensity is too loud when the image is collected, and the acquisition device has a focus on this area, which has led to this kind of phenomenon of uneven illumination.
(4) The effect of different algorithms to deal with the uneven illumination of the QR code with higher overall contrast is shown in Figure 13. The cause of uneven illumination is that the image is obscured, which results in partial shadows on the image.
(5) The effect of different algorithms to deal with the uneven illumination of the QR code with lower local contrast is shown in Figure 14. This phenomenon occurs when the local illumination is too intense.

**Figure 15:**
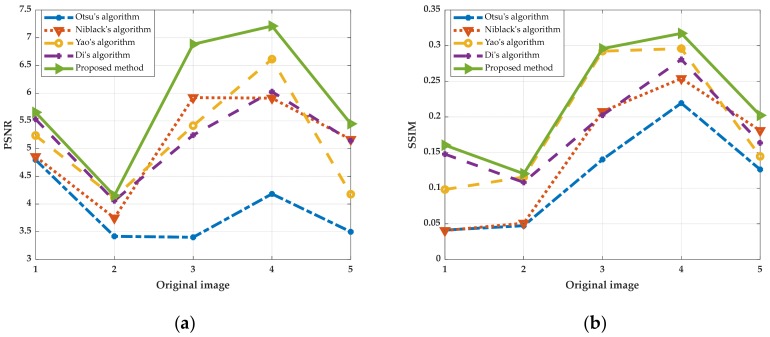
The values of PSNR and SSIM of different QR codes using different algorithms. (**a**) The line graph corresponding to PSNR; (**b**) The line graph corresponding to SSIM.

The values of PSNR and SSIM obtained by binarization of different uneven illuminations of the QR code images after the proposed method and other algorithms are listed in Table 4, and the broken line graph is shown in Figure 15.

Clearly, the proposed method has a better effect than the other comparison algorithms when dealing with multiple uneven illumination QR image types from Figure 10 to Figure 14. Especially in the case of over-illuminated and obscured QR code images, using the Otsu algorithm directly will cause large pieces of information to disappear, while using the Niblack’s algorithm will generate a lot of noise. Even though the noise can be eliminated by adjusting the size of the block window, the algorithm needs to test to find the appropriate window constantly and the operation time is too long. Yao’s algorithm is suitable for the binarization of QR code images with no intense illumination changes and no apparent change boundaries. When the illumination changes sharply at the boundary, the algorithm cannot classify the pixels in the boundary buffer region well, which leads to the reduced effect of binarization. Di’s algorithm causes black block or missing information at the position where the light intensity changes drastically. What is more, according to the information in Table 4 and Figure 15, the proposed algorithm is superior to other the comparison algorithms in PSNR and SSIM. It illustrates that the image binarized by the proposed method has a higher quality, which proves that the proposed method has consistent performance in subjective and objective evaluation.

Furthermore, several algorithms are compared for operation time and recognition rates. There are 80 experimental samples of QR code images under different illumination conditions, including 30 images with weak uneven illumination and 50 images with intense uneven illumination. The resolution of the image is 300 pixels × 300 pixels. Then, the barcode recognition software ZXing AndroidSDK and WeChat are used to decode and identify the binarized image, respectively. The comparison results are shown in Table 5.

The analysis of Table 5 reveals that the speed of Otsu’s algorithm and Yao’s algorithm is faster, but the effect is not good enough, and even the recognition rate of Otsu’s algorithm is lower than that without the algorithms. The proposed algorithm determines the size of the block window by pre-processing, which shortens the time for the algorithm to traverse the image. Thus, the operation speed of the algorithm is improved greatly. Compared with the Niblack’s algorithm and Di’s algorithm, the proposed algorithm has a higher average calculation efficiency and recognition rate.

To summarize, compared with other algorithms, the experimental results demonstrate that the proposed method has better performance in QR code recognition and its binarization results and effectiveness are superior to other approaches.

## 5. Conclusions

An adaptive binarization approach based on QR code symbol features was proposed to solve the problem of QR code scanning under uneven illuminations in warehouse management applications. The algorithm automatically calculates the size of the image block according to the QR code position detection pattern, based on which binarization is applied block-by-block. Intrinsically, we successfully solved the problem and helped to extract a complete QR code, even when the illumination varied unevenly, particularly for the binarization of the uneven illumination of the QR code under intense local illumination and obscured image. The algorithm is suitable for QR code binarization in complex illumination environments, such as warehouse auto-sorting systems, even in a real-time manner. Intensive experiments have fully validated the efficacy and robustness of the proposed approach.

## Figures and Tables

**Table 1:** Hardware equipment.

Hardware	Parameters
Computer	CPU: Intel (R) Core i7-8700 3.2GHz OS: Window10 Education Edition Display: HP V220 Resolution: 1920×1080 dpi
Mobile phone	Model: Xiaomi 6 Rear camera pixel: 12 million
Laser printer	Model: HP M226dw Printed resolution: 600×600 dpi

**Table 2:** Software environment.

Software	Version	Language
MATLAB	R2018a	Simplified Chinese
ZXing AndroidSDK	Zxing-3.1.0.jar	
WeChat	Vesion7.0.4	Simplified Chinese

**Table 3:** Other experimental materials.

Materials	Parameters
Captured images	Type: QR code Format: jpg Bits: 24
Printed paper, Toner	80g/m^2^ A4 Paper, NT-C0388CT

**Table 4:** The values of PSNR and SSIM of different QR codes using different algorithms.

Type	Image Quality	Otsu’s Algorithm	Niblack’s Algorithm	Yao’s Algorithm	Di’s Algorithm	Proposed Method
(1)	PSNR	4.7978	4.8562	5.2366	5.5307	5.6547
	SSIM	0.0413	0.0406	0.0982	0.1478	0.1604
(2)	PSNR	3.4180	3.7501	4.1174	4.0594	4.1494
	SSIM	0.0474	0.0509	0.1157	0.1082	0.1204
(3)	PSNR	3.4005	5.9200	5.4138	5.2435	6.8823
	SSIM	0.1404	0.2076	0.2923	0.2025	0.2959
(4)	PSNR	4.1827	5.9107	6.6141	6.0277	7.2117
	SSIM	0.2194	0.2539	0.2959	0.2807	0.3172
(5)	PSNR	3.4994	5.1656	4.1776	5.1406	5.4483
	SSIM	0.1263	0.1809	0.1447	0.1636	0.2022

**Table 5:** Comparison of average calculation time and recognition rate of five algorithms.

Algorithm	Average Operation Time/s	Recognition Rate	
		Zxing	WeChat
**None**	——	35%	48.75%
Otsu’s algorithm	0.0013	32.5%	32.5%
Niblack’s algorithm	1.6063	38.75%	86.25%
Yao’s algorithm	0.0046	37.5%	42.5%
Di’s algorithm	0.3516	53.75%	71.25%
Proposed method	0.0428	88.75%	92.5%
